# Case Report: Surgical treatment of feline pituitary apoplexy in 3 cases

**DOI:** 10.3389/fvets.2026.1779079

**Published:** 2026-10-09

**Authors:** Megan A. van Schaik, Lucinda L. van Stee, Guy C. M. Grinwis, Björn P. Meij

**Affiliations:** 1 Department of Clinical Sciences, Utrecht University, Utrecht, Netherlands; 2 Veterinary Pathology, Department Biomolecular Health Sciences, Faculty of Veterinary Medicine Utrecht University, Utrecht, Netherlands

**Keywords:** apoplexy, cat, hypophysectomy, intracranial hemorrhage, intracranial infarction, pituitary, transsphenoidal

## Abstract

**Background:**

Surgical treatment of feline pituitary apoplexy has not been previously described.

**Objectives:**

To describe the clinical course and follow-up of 3 cases of feline pituitary apoplexy.

**Methods:**

Cases were selected based on histopathological confirmation of pituitary masses with haemorrhagic lesions, following transsphenoidal hypophysectomy. Details of physical examination, diagnostic test results, treatment and follow-up course were retrospectively acquired by review of medical and imaging records.

**Results:**

Two cats were presented with acute onset of neurologic signs (case 1 and 2), and one cat showed subacute endocrinological dysfunction (case 3). All cases underwent successful transsphenoidal hypophysectomy, followed by intensive care hospitalization and successful discharge. Pituitary apoplexy was identified by histopathological examination of the excised pituitary glands in cases 1, 2, and 3. Imaging findings compatible with pituitary apoplexy were present in all 3 cases, although only case 3 was imaged with a hemorrhage-sensitive sequence, providing unequivocal evidence of intralesional hemorrhage. Case 1 had a survival time at least 716 days, underwent a second transsphenoidal surgery because of tumor recurrence and was subsequently lost to follow-up. Case 2 had a survival time of at least 1,098 days and is still alive at the time of writing this manuscript. In case 3, tumor recurrence was identified radiologically (MRI) at 365 days, and it died during a course of radiation therapy 612 days after transsphenoidal hypophysectomy.

**Conclusion and clinical importance:**

Pituitary apoplexy is a rare condition that also affects cats with large pituitary adenomas. Overall, the cats experienced acceptable clinical recovery and postoperative survival, supporting the feasibility of transsphenoidal hypophysectomy as a therapeutic option for cats with pituitary tumors affected by apoplexy. Notably, a favorable outcome was observed even in the presence of remnant pituitary tissue, as illustrated by the cat that later developed pituitary tumor recurrence.

## 1 Introduction

In human medicine, pituitary apoplexy (PA) is characterized by a sudden onset of severe headaches, altered levels of consciousness and visual impairment originating from a sudden increase in intrasellar pressure, caused by hemorrhage and/or infarction of the pituitary gland. This condition generally arises in patients with pre-existing non-functional pituitary macroadenomas, but it can also develop in individuals with healthy pituitary glands, or patients affected by inflammation or infection (1). Diagnosis is based on clinical suspicion, in combination with imaging, where magnetic resonance imaging (MRI) is the preferred option. Findings consistent with PA include signs of hemorrhage, necrosis or oedema, often in association with a pituitary neuroendocrine tumor (2). Therapeutic options consist of either early transsphenoidal decompression or conservative approach for patients without neuro-ophthalmic signs or with mild and stable symptoms (3).

In veterinary literature, and especially in cats, PA is scarcely described. In the feline cases that have been documented, episodes of restlessness, acute blindness, disorientation, and depression have been observed. Diagnoses were based on radiological findings in combination with histopathology. In these cases, no surgical intervention was performed, and the cats were euthanized (4–6).

This report describes the first cases of cats with pituitary apoplexy, which underwent successful transsphenoidal hypophysectomy (THX) and recovery. Initially, all cases were diagnosed with a pituitary mass, based on radiological examination. All 3 cases showed a heterogeneous pituitary gland on advanced imaging, indicative of hemorrhage, infarction or necrosis. Subsequently, they underwent hypophysectomy, indicated by the large P/B ratios, neurological or endocrinological manifestations. The definitive diagnoses of pituitary apoplexy were based on histopathological examination. Pituitary apoplexy is a differential diagnosis that should be given greater consideration in patients with an acute onset of neurological signs, as outlined above, to optimize diagnostic accuracy and thereby improve outcomes for affected patients. Surgical intervention in the cases described in this case report led to a swift neurological recovery and positive long-term outcome.

## 2 Case descriptions

### 2.1 Case 1

An 11-year-old neutered female Domestic Shorthaired cat was presented with a 2-year history of hyporexia and progressive weight loss of 2 kg (from 6.7 to 4.7 kg), as well as a 2-week history of acute behavioral abnormalities. Clinical signs included aimless pacing along walls in both directions, restlessness and aggression toward other cats. The cat was also actively seeking contact toward the owners. General physical and neurological examinations of this cat were unremarkable; the aforementioned changes in behavior could not be verified during the consultation. No endocrine disease was diagnosed, based on serum thyroxine (T4) concentrations of 29 nanomole/L (nmol/L) (15–45), thyroid-stimulating hormone concentrations < 0.03 microgram/L (mcg/L) (< 0.03 mcg/L), insulin-like growth factor-1 (IGF-1) concentrations of 268 mcg/L (< 598) and adrenocorticotropic hormone (ACTH) concentrations of 64 picogram/mL (pg/mL) (–358) (7, 8).

Computed tomography (CT) demonstrated a markedly enlarged pituitary gland measuring 8.7 mm (*H*) × 15.1 mm (*L*) × 10 mm (*W*) and a pituitary height-to-brain area (P/B) ratio of 0.96. Although no validated feline reference value for the P/B ratio is available, the pituitary height exceeded the 4.0 mm threshold used to define pituitary enlargement in cats (9), and the P/B ratio was well above the canine cut-off of 0.31 (10). Additionally, intralesional hyperattenuating and hypoattenuating areas were also present, suggestive of haemorrhagic and infarctive changes within the mass (Figures 1A, B). Considering the size of the tumor and the favorable outcomes reported for THX in cats, surgical intervention was elected as the treatment of choice in this case.

**Figure 1:**
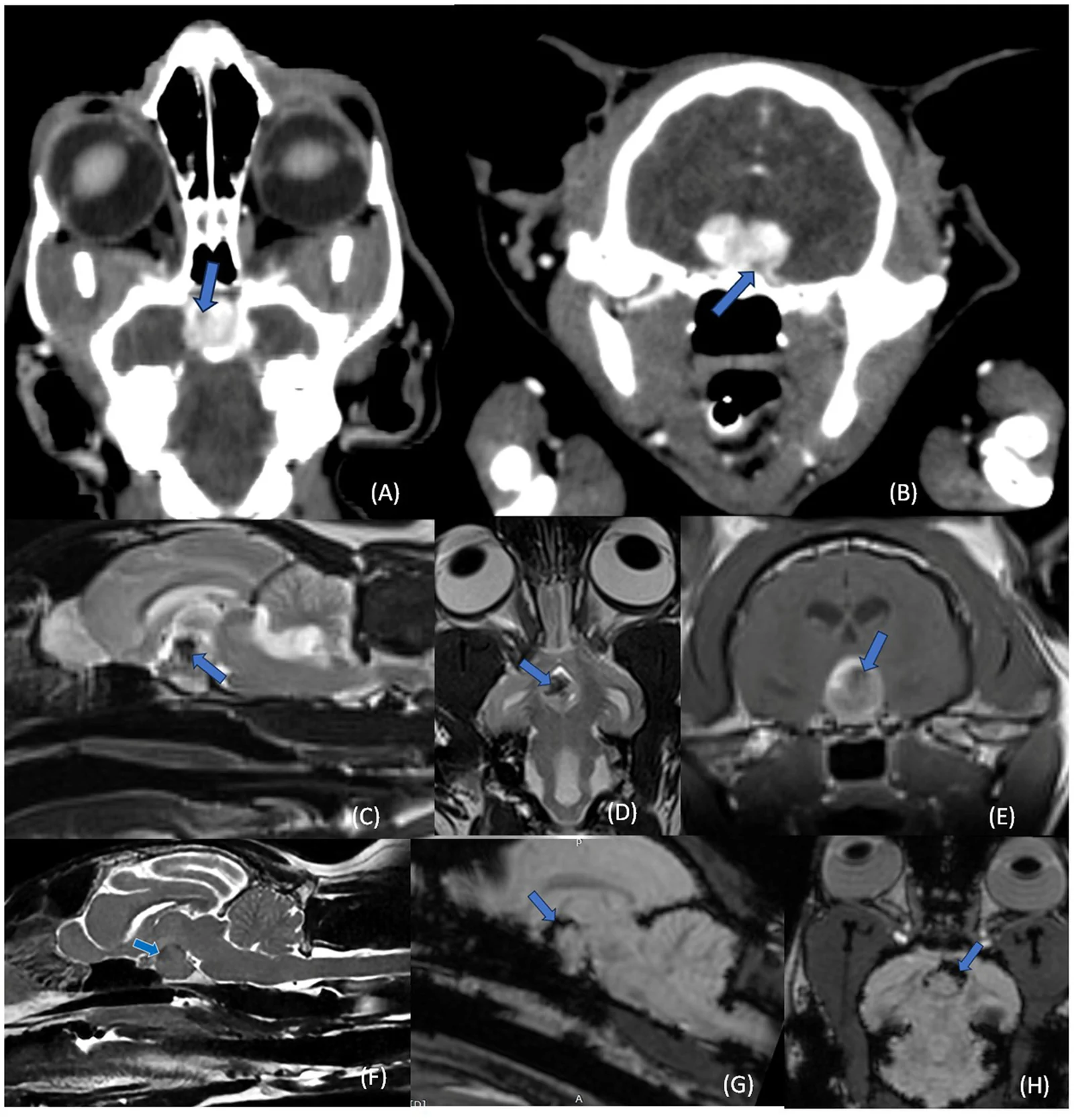
Preoperative imaging. **Case 1:** Post contrast computed tomography (CT) dorsal **(A)** and transverse **(B)** reconstructions showing a very large pituitary mass (P/B 0.96), characterized by marked and diffuse hyperattenuating and a single well-defined hypoattenuating area (blue arrow), which can be suggestive of hemorrhage or necrosis. **Case 2**: T2-weighted MRI sagittal **(C)** and dorsal **(D)** images and T1-weighted post-contrast MRI transverse **(E)** image demonstrating a large pituitary mass (P/B 1.25) with intralesional hypointense areas in T1w and T2w (blue arrows) suggesting intralesional hemorrhage. **Case 3:** Sagittal T2-weighted **(F)**, sagittal T2-weighted SWAN **(G)**, and dorsal T2-weighted SWAN **(H)** MRI images showing an enlarged pituitary mass (P/B 1.12), that contains hypointense peripheral (blue arrows) regions in both sequences compatible with blooming artifact, which is compatible with intralesional hemorrhage.

The day prior to surgery, the cat was hospitalized for pre-operative stabilization. Intravenous fluid therapy (IVFT) was administered at a maintenance rate of 8 ml/h. Analgesia was provided with meloxicam 0.1 mg/kg one time daily (SID). A nasal feeding tube was placed to enable nutrition intake (Royal Canin Convalescence Support).

Prior to THX, the cat was pre-medicated with 0.70 mg (0.15 mg/kg) methadone hydrochloride intravenously (IV) and induced smoothly with 1.08 mg (0.22 mg/kg) midazolam IV and 15 mg (3.1 mg/kg) propofol IV. It was intubated with an endotracheal tube size 4, directly followed by manual ventilation (EtCO2 4–5 kPa). Anesthesia was maintained using sevoflurane, remifentanil 0.2–0.3 mcg/kg/min IV Constant Rate Infusion (CRI) and propofol 0.5–1 mg/kg IV bolus on effect. Local infiltration of the incision site using lidocaine 14.4 mg (3.06 mg/kg). Monitoring consisted of invasive and non-invasive arterial blood pressure, capnography, electrocardiogram (ECG), FiO2, pulsoximetry and spirometry. Body temperature was supported using Bairhugger and a warming matrass. Intra-operative bradycardia treated on effect with atropine 3–5 mcg/kg IV and glycopyrrolate 5 mcg/kg IV. Intra-operative hypotension treated on effect with dopamine 3–6 mcg/kg/min IV CRI. Reduction of intracranial pressure by maintaining CO_2_ levels < 4.0 mmHg.

THX was performed as described in a previous publication on this procedure in cats (9). The soft palate was incised in the midline using a No. 10 scalpel blade and retracted laterally with a self-retaining Gelpi retractor. The mucoperiosteum was separated from the sphenoid bone and the hamular processes. Subsequently, the sphenoid ridge was identified, to determine the position of the burr hole. A 10 mm window was created in the sphenoid bone, using a burr. Rostral perforation into the air-filled sphenoid sinus identified the position of the fossa, located caudally to this point. The approach was then continued caudally directly toward the pituitary gland. Bleeding from the bone was controlled with bone wax (Ethicon, Somerville, NJ, USA). In the cancellous portion of the bone, haemostasis was achieved with bone wax. The opening measured approximately 10 mm in length and 8 mm in width. The pituitary gland appeared red-blue rostrally, suggestive of tumor, and white caudally, suggestive of normal tissue. The dura mater was coagulated using bipolar electrocautery and incised using a No. 11 blade. The apparent abnormal tissue was carefully dissected free from surrounding tissue and removed in multiple fragments, which were all submitted in a single formalin container, including some macroscopically normal-appearing pituitary tissue. The fossa was emptied and the dorsum sellae was palpable. The fossa was filled with a gelatine foam sponge (Spongostan Absorbable Hemostatic Gelatin Sponge (Ethicon, Somerville, NJ, USA), approximately 8 × 6 mm. Bone wax (Ethicon, Somerville, NJ, USA) was applied to the sphenoid defect and mucoperiosteum. Caudally, a single suture of polydioxanone (PDS II 6-0, Ethicon, Somerville, NJ, USA) was placed with good apposition of the mucoperiosteum. Bone wax coverage was approximately 50%. The soft palate was closed in two layers; nasal mucosa was sutured using polygalactin 910 (Vicryl 4-0, Ethicon, Somerville, NJ, USA), in a simple continuous pattern, and the oral mucosa was sutured using polyglactin 910 (Vicryl 4-0, Ethicon, Somerville, NJ, USA) in a simple interrupted pattern. The recovery was smooth and uneventful.

The cat was admitted to the intensive care unit for postoperative monitoring. General physical examination (respiratory rate (RR), heart rate (HR), temperature, mucus membranes and capillary refill time (CRT) was performed every 4 h. Plasma sodium, potassium, glucose and haematocrit (Ht) levels were measured 2 times daily (BID). Urine production and fluid balance was monitored and calculated every 2 h. The IVFT maintenance rate was 8 ml/h (Sterofundin B/G) and corrections were administered accordingly. To maintain nutrition intake, the cat had received an esophageal feeding tube. Royal Canin Convalescence Support was administered at a CRI. Analgesia was provided with buprenorphine [20 mcg/kg 3 times daily (TID) IV] and antibiotic prophylaxis was initiated with amoxycillin-clavulanate (20 mg/kg TID IV). The cat received hormone replacement therapy with hydrocortisone IV [1 mg/kg 4 times daily (QID)], desmopressin ocular drops TID, alternating the right eye (OD) and left eye (OS), and oral thyroxine (15 mcg/kg BID). The cat was severely bradycardic (100–130 bpm) and hypotensive, had mild hyponatremia [135 mmol/L (146–158 mmol/L)] and was severely anemic [Ht: 0.14 L/L (0.28–0.47 L/L)], for which the cat received a blood transfusion, correcting the Ht to 0.19. During this period, bilateral mydriasis was observed, with absent direct and consensual pupillary reflexes and an absent menace response in both eyes. Meanwhile there were no detectable intraocular abnormalities. Examination by a board-certified veterinary ophthalmologist confirmed bilateral blindness, considered most likely to be related to the surgical procedure. A Schirmer Tear Test (STT) also identified keratoconjunctivitis sicca (KCS), measuring a tear production of 0 mm/min in both eyes (OU). After 8 days, the cat was stable and discharged for appropriate aftercare at home. Life-long hormone replacement therapy consisted of a maintenance dose of oral cortisone acetate (0.35 mg/kg BID), oral L-thyroxine (15 mcg/kg BID). Diabetes insipidus was managed by administering desmopressin eyedrops BID, alternating right eye (Oculus Dexter, OD) and left eye (Oculus Sinister, OS), measuring plasma electrolyte levels after 1 and 4 weeks, and adjusting the dose accordingly. Once normal plasma electrolyte levels were achieved, treatment with the ocular drops was continued for an additional week, after which they could be discontinued. The visual impairment remained unchanged for the remainder of the cat's life.

After 3 months, a CT scan of the skull was repeated to monitor for recurrence of pituitary tumor. Changes within the pituitary tissue were compatible with a minute amount of residual hypophyseal/neoplastic tissue, although these findings were not conclusive. Clinical follow up, with repeat CT imaging if deemed indicated, was recommended after 6–12 months and annually thereafter.

At 705 days after the initial surgery, recurrence was diagnosed through repeat imaging (CT), with the following dimensions: 7.8 mm (*H*) × 9.7 mm (*L*) × 6.7 mm (*W*) and a pituitary/brain ratio (P/B) of 1.0. The cat underwent a second THX. Following THX, the cat remained in the ICU for 11 days before being discharged. The cat was lost to follow-up after discharge at a registered survival time of 716 days.

The histopathological and immunohistochemical (IHC) evaluation of the excised tissue showed pituitary apoplexy and a basophilic tumor that demonstrated marked absence of both ACTH and GH staining in the IHC in the first instance. Tumor tissue obtained during the second surgical procedure showed an even more homogenic basophilic tumor cell population, with again, no immunoreactivity for ACTH and GH.

### 2.2 Case 2

A 12-year-old neutered male European Shorthaired cat was presented with neurologic signs. The cat exhibited anorexia for 1-month, acute episodes of weakness in the hind limbs, and decreased mentation. General examination showed a quiet and responsive cat with a markedly reduced general condition. The neurologic examination revealed a minimally delayed direct bilateral pupillary reflex, while corneal and palpebral reflexes were intact. There was a marked reduced sensitivity of the nose. The cat showed delayed visual and absent tactile proprioception in the thoracic limbs. Proprioception was normal on the left front limb, while markedly delayed on the right front limb, although this was the leg wrapped with a catheter bandage dressing. Dorsiflexion of the neck elicited marked ventral strabismus in the left eye and vertical nystagmus in both eyes. There were no signs of endocrinopathy like diabetes mellitus (DM) or polyuria/polydipsia (PUPD).

A sonographic examination of the heart was performed as well, which showed no structural cardiac abnormalities or function impairment. Cerebrospinal fluid (CSF) and plasma were assessed on Toxoplasmosis. CSF analysis revealed IgG 94.16 (< 50), IgM 4.45 (< 9) and total protein 1.01 g/L. Plasma serology showed IgG 203.39 (< 50), IgM 18.54 (< 9). IGF-1 (399 ng/mL, < 598), basal serum cortisol [12.5 ng/mL (2–50 ng/mL)] were within reference range and FeLV / FIV testing was negative. CSF total nucleated cell count, cytology and antigen titres were not performed at the time.

MRI was performed, which identified a large round pituitary mass [12.2 mm (*H*) × 10.8 mm (*L*) × 11.2 mm (*W*)] with a P/B ratio of 1.25. The mass compressed the thalamus and brainstem (caudal displacement), and moderate-marked congestion of both lateral ventricles and the fourth ventricle were observed. T2-weighted and T1-weighted images demonstrated hypointense areas within the mass, suggestive of intralesional hemorrhage (Figures 1C–E). The spinal cord of the cervical spine showed a marked syringomyelia. Like case 1, THX was selected as the treatment of choice because of the large size of the pituitary mass and the favorable outcomes reported following this procedure.

The day prior to surgery, the cat was hospitalized for preoperative stabilization. Complete blood cell count and routine biochemical analysis were performed. A stress leucogram was noted, and mild increase in total protein and albumin. ACTH was within normal limits [215 pg/mL (15–358 pg/mL)]. The cat received hydrocortisone 1 mg/kg, amoxicillin-clavulanate intravenously IV [20 mg/kg 3 times daily (TID)], maropitant IV (1 mg/kg SID), omeprazole IV (1 mg/kg SID) and gabapentin (15 mg/kg once). A feeding tube and jugular catheter were placed. On the day of surgery, a repeat Toxoplasma gondii serology was performed at a second laboratory, 3.5 weeks after the positive serology and CSF titres obtained at initial presentation. The combined IgG and IgM titer was 1:256 (reference < 1:64).

Prior to surgery, the cat was premedicated with methadone-hydrochloride 1.66 mg (0.25 mg/kg) IV and dexmedetomidine 13.4 mcg (2 mcg/kg) IV, providing a mild sedation. The cat was induced with propofol on effect. It was intubated with an endotracheal tube size 3.5. Anesthesia was maintained using isoflurane, sufentanil 0.5–1 mcg/kg/min IV CRI and ketamine 10 mcg/kg/min IV CRI. Local infiltration of the incision site using lidocaine 13.2 mg (2 mg/kg). Monitoring adhered to the same protocol as outlined in case 1, with the addition of arterial blood gas analysis. Intra-operative bradycardia treated on effect with atropine 5 mcg/kg IV and glycopyrrolate 1–10 mcg/kg IV. Intra-operative hypotension treated on effect with dobutamine 0.55–8 mcg/kg/min IV CRI. Reduction of intracranial pressure by maintaining CO_2_ levels < 4.0 mmHg, and administration of mannitol (0.25 g/kg over 20 min).

THX performed as described in case 1. Upon opening the pituitary fossa, the bulging pituitary gland was rapidly encountered. The tissue showed a heterogenous aspect. Apparent abnormal tissue (mucoid and gray) was removed from the fossa in multiple fragments and submitted in a formalin container. The surgeon observed macroscopic complete excision. Throughout the surgical procedure, the approach and fossa contents were examined using a 18 cm 1.9 mm 30° forward telescope, confirming complete extirpation (Figures 2A, B). Closure of the surgical side was performed as described in case 1.

**Figure 2:**
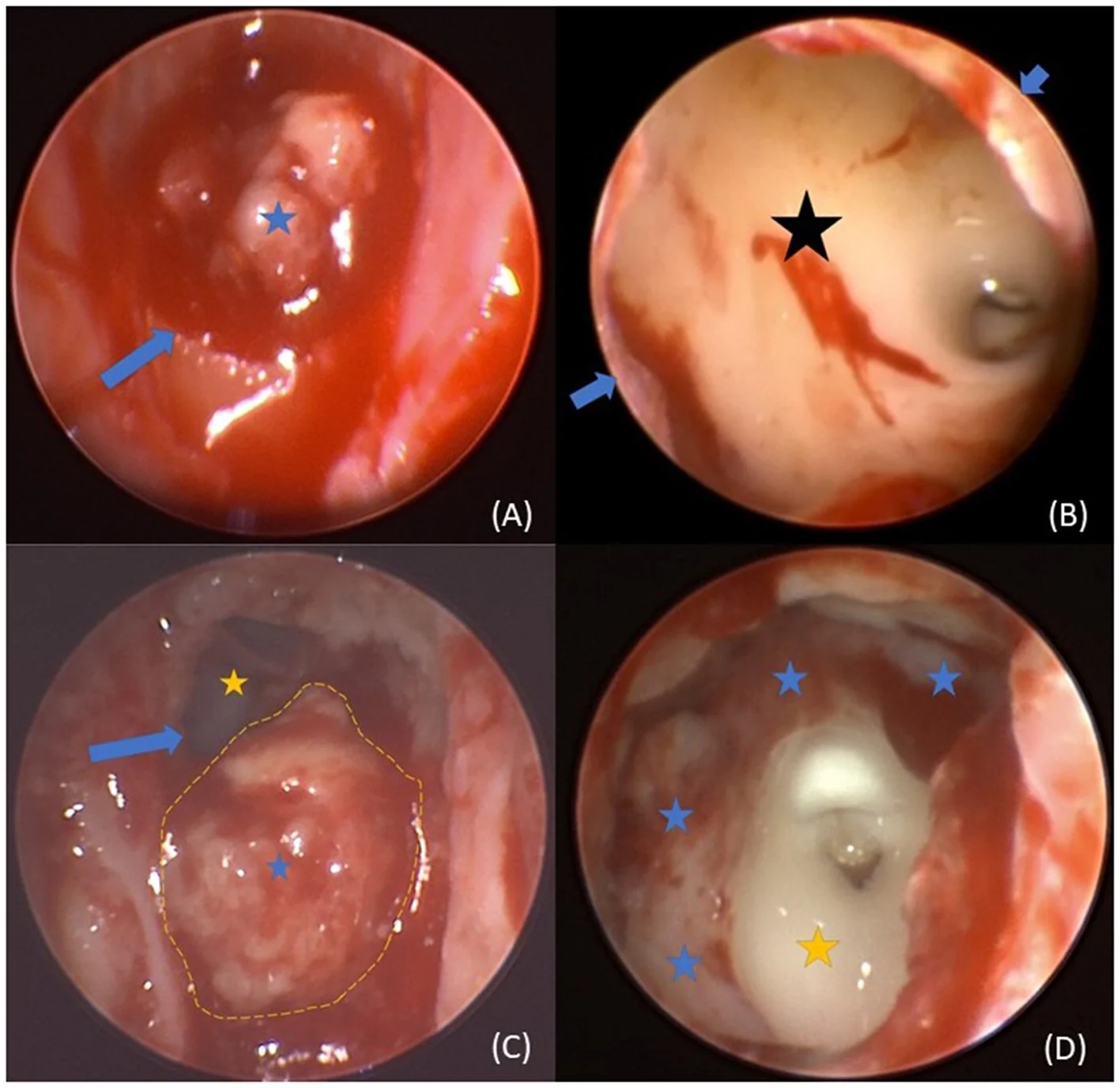
Intraoperative endoscopy. **Case 2: (A)** Intraoperative endoscopic image of the pituitary mass (blue star) bulging through the sphenoidotomy (blue arrow). **(B)** Endoscopic view of the pituitary fossa after complete mass extraction. The edges of the sphenoidotomy are marked by blue arrows, and a clear view of the hypothalamus (black star) is obtained. **Case 3**: **(C)** Intraoperative endoscopic view of the pituitary mass (yellow dotted line, blue star) protruding through the sphenoidotomy (blue arrow). In the background, the hypothalamus is already visible (yellow star). **(D)** During tumor extraction, the hypothalamus becomes visible (yellow star) while there is still remnant tumor tissue (blue stars) present in the periphery.

A direct post-operative CT scan of the head was performed and compared to the preoperative MRI, confirming absence of the previously visualized mass. The remaining brain parenchyma was unremarkable. There was a marked decompression of the thalamus and brainstem.

After surgery, the cat recovered swift and without complications. Subsequently, the cat was admitted to the ICU, where it had a hospitalization period of 6 days. Postoperative medication, monitoring, and hormone replacement therapy were followed as protocolized and described in case 1. Blood glucose level was measured every 4 h, which remained < 9 mmol/L throughout the hospitalization period, without the administration of insulin. This indicated a remission of DM. Furthermore, cat received clindamycin 12.5 mg/kg BID, indicated by positive Toxoplasma plasma titres. A STT identified KCS, with values between 5 and 6 mm/min. in both eyes. During this period, the cat exhibited adipsia and anorexia, for which the cat received nutrition through an esophageal feeding tube 5 times daily. Furthermore, the cat developed moderate hyperthermia (40.4 °C), which resolved without additional therapy after 3 days. After 6 days, the cat was considered stable and discharged with appropriate aftercare at home following protocol as described in case 1.

Histopathological and IHC examination of the excised tumor tissue showed a basophilic and ACTH positive profile on IHC staining and pituitary apoplexy.

Two weeks after surgery, the cat was examined by the referring veterinarian. The food and water intake increased significantly over time, but in general the cat was still in slight lethargic state. Further general physical examination was unremarkable although the KCS persisted. The cat is still alive at the time of writing of the manuscript. Although the lethargic state reportedly never fully resolved, there has been no development of new or recurrent neurological signs, resulting in a disease-free interval of 1,098 days. The owner was encouraged to have follow up advanced diagnostic imaging of the pituitary fossa performed on the cat at 6- and 12-months post-surgery followed by yearly imaging rechecks. However, they declined and follow up has been performed through predominantly email contact.

### 2.3 Case 3

A 9-year-old neutered male Domestic Shorthaired cat was presented with increased appetite, weight gain (from 5.1 kg to 5.7 kg), locomotor difficulties due to muscle wasting and PUPD at the referring veterinarian. DM was confirmed with detection of glucosuria and persisting elevated blood glucose levels. Insulin therapy was initiated at a dose of 4.5 IU of insulin (Caninsulin^®^, Intervet, Boxmeer, The Netherlands) BID administered subcutaneously (SC). PUPD improved but did not completely resolve. Subsequently, the cat developed spontaneous skin fragility, leading to suspicion of Fragile Skin Syndrome, which can be caused by hypercortisolism. An excessive response to an ACTH stimulation test was observed, and ultrasonography revealed bilateral adrenal enlargement.

CT and MRI identified a rounded pituitary mass with the following dimensions: 10 mm (*H*) × 10 mm (*L*) × 11 mm (*W*), P/B ratio of 1.12. T2-weighted MRI images were characterized by hypointense regions of hemorrhage (Figure 1F). T2-weighted SWAN images demonstrated a hypointense signal with a blooming artifact, which is very indicative of hemorrhage (Figures 1G, H) (11). Based on the large tumor size and the presence of endocrinopathy, surgery was the elected treatment of choice in this cat.

With the presumptive diagnosis of pituitary-dependent hypercortisolism (PDH), the cat was started on trilostane (Vetoryl, Dechra Regulatory B.V., Bladel, the Netherlands). Over a period of 2 months, the dosage was increased from 5 mg to 10 mg to 30 mg, BID. Serum IGF-1 or growth hormone (GH) was not measured, therefore, hypersomatotropism (HS) was not ruled out in this cat prior to surgical intervention.

One day before surgery, the cat was hospitalized and was prepared for surgery as described in case 1. Blood glucose concentrations were measured every hour, and insulin was adjusted accordingly.

Prior to surgery, the cat was premedicated with methadone-hydrochloride 1.71 mg (0.3 mg/kg) IV and dexmedetomidine 11.4 mcg (2 mcg/kg) IV, providing a mild sedation. The cat was induced with propofol on effect. It was intubated with an endotracheal tube size 3.5. Anesthesia was maintained using isoflurane and dexmedetomidine CRI at 0.6–1.0 mcg/kg/h. Local infiltration of the incision site using lidocaine 11.4 mg (2 mg/kg). Monitoring adhered to the same protocol as outlined in case 1, with the addition of arterial blood gas analysis. Additionally, plasma glucose concentrations were monitored every 10 min, and insulin CRI was adjusted accordingly. Reduction of intracranial pressure was achieved by maintaining CO_2_ levels < 4.0 mmHg. THX was performed, as described in case 1. During the THX, the complete pituitary gland was removed including the apparent abnormal adenohypophysis, as macroscopically observed by the surgeon. The extracted affected tissue contained haemorrhagic and gray mucoid areas, which were collected for histological examination. During the procedure, the surgical approach and content of the fossa were examined endoscopically using a 18 cm 1.9 mm 30° forward angled telescope (Figures 2C, D). Endoscopy provided a better visualization of the pituitary fossa content, enabling resection of tumor tissue that lies in the periphery of the burr hole (Figure 2D). Endoscopy confirming absence of residual pituitary tissue in the fossa. Closure of the surgical side was performed as described in case 1.

Post-operative head CT was performed, demonstrating absence of the pituitary gland. However, contrast enhancement in the right caudal portion of the brain parenchyma (±4 x 4.5 x 3.2 mm), suggested possible incomplete resection of the pituitary gland.

The cat underwent a swift recovery. The cat was admitted to the ICU after hypophysectomy and was treated with the standardized protocol as described in case 1. During the hospitalization, mydriasis was observed in both eyes after initial good recovery from anesthesia. The apparent blindness was noted directly after buprenorphine and hydrocortisone administration, and was combined with extreme excitation. After examination by an ophthalmologic specialist the following day, the cat was identified with corneal ulcerations and impaired vision. Direct and consensual pupillary reflexes were absent in both eyes (OU), however, menace and dazzle reflexes were both present. Concurrent opioid administration was considered a possible contributing factor for the absence of pupillary reflexes at the time, although this could not be determined with certainty. Tear production was within reference range in both eyes. No abnormalities were seen during fundoscopy. The visual impairment was considered most likely secondary to surgical trauma, possibly cortical in nature as mydriasis and possible subsequent inadequate pupillary reflexes were attributed to the response to opioids at the time; however, the exact etiology could not be definitively determined. The cat was discharged 7 days after surgery, with complete resolution of DM. Over time, in the months following surgery, vision appeared to improve partially. Mydriasis persisted, although not as profound as it was during postoperative hospitalization.

Histopathological and IHC analysis of the pituitary tissue confirmed the presence of a basophilic and ACTH positive tumor, combined with pituitary apoplexy.

After hypophysectomy, remission of Cushing's disease was monitored. Direct post-operative ACTH evaluations demonstrated a marked decrease in plasma ACTH concentrations (pre-THX: 877 pg/ml, post THX *T* = 1 h: 298 pg/ml, post THX *T* = 3 h: 176 pg/ml, post THX *T* = 5 h: 156 pg/ml). Basal urinary cortisol/creatinine ratios (UCCR) have been measured multiple times after surgery. Respectively after discharge, 3 weeks, 8 weeks, 6 months and 1-year UCCRs were 8.9, 28.5, 6.7 and 11.5. All measured concentrations were within reference range (2–36) (7). MRI follow up was performed at approximately 12 and 18 months after surgery, in which local tumor recurrence was first noted on the 12 months MRI, with a mass measuring approximately 5 x 6 x 4 mm, located on the caudal left-hand side of the pituitary fossa. At that time, no clinical signs involving endocrinological nor neurological disease were noted. At 18 months, tumor mass slowly progressed measuring 6 x 7 x 7 mm on the MRI images, without recurrence of the preoperative signs of diabetes mellitus or hypercortisolism.

Due to this presence of tumor mass in the pituitary fossa detected on the 12-month post-surgery MRI and slow progression on the 18-month post-surgery MRI, the cat underwent curative intent external beam radiotherapy (RT) at the time of the reviewing process of the manuscript. Although a second surgical procedure was possible, with the current absence of endocrinological disease, the fractious nature of the cat and the local availability of RT, this minimally invasive therapy was chosen. The cat died at home during the treatment period due to unrelated comorbidities at 612 days post THX. No postmortem examination was performed.

## 3 Histology

Histopathological examination was performed by a board-certified veterinary pathologist (GG). Routine haematoxylin and eosin (H&E) staining, in combination with immunohistochemical staining for ACTH, GH, and alpha-melanocyte-stimulating hormone (α-MSH), was performed in all cases.

Case 1 (Figures 3A–D) demonstrated a basophilic adenoma, while it stained negative for ACTH, GH and alpha-MSH on immunohistochemistry in both tissue samples obtained during the two consecutive surgeries. However, the recurrent tumor tissue showed a more monotonous population of neoplastic cells compared to the primary tumor.

**Figure 3:**
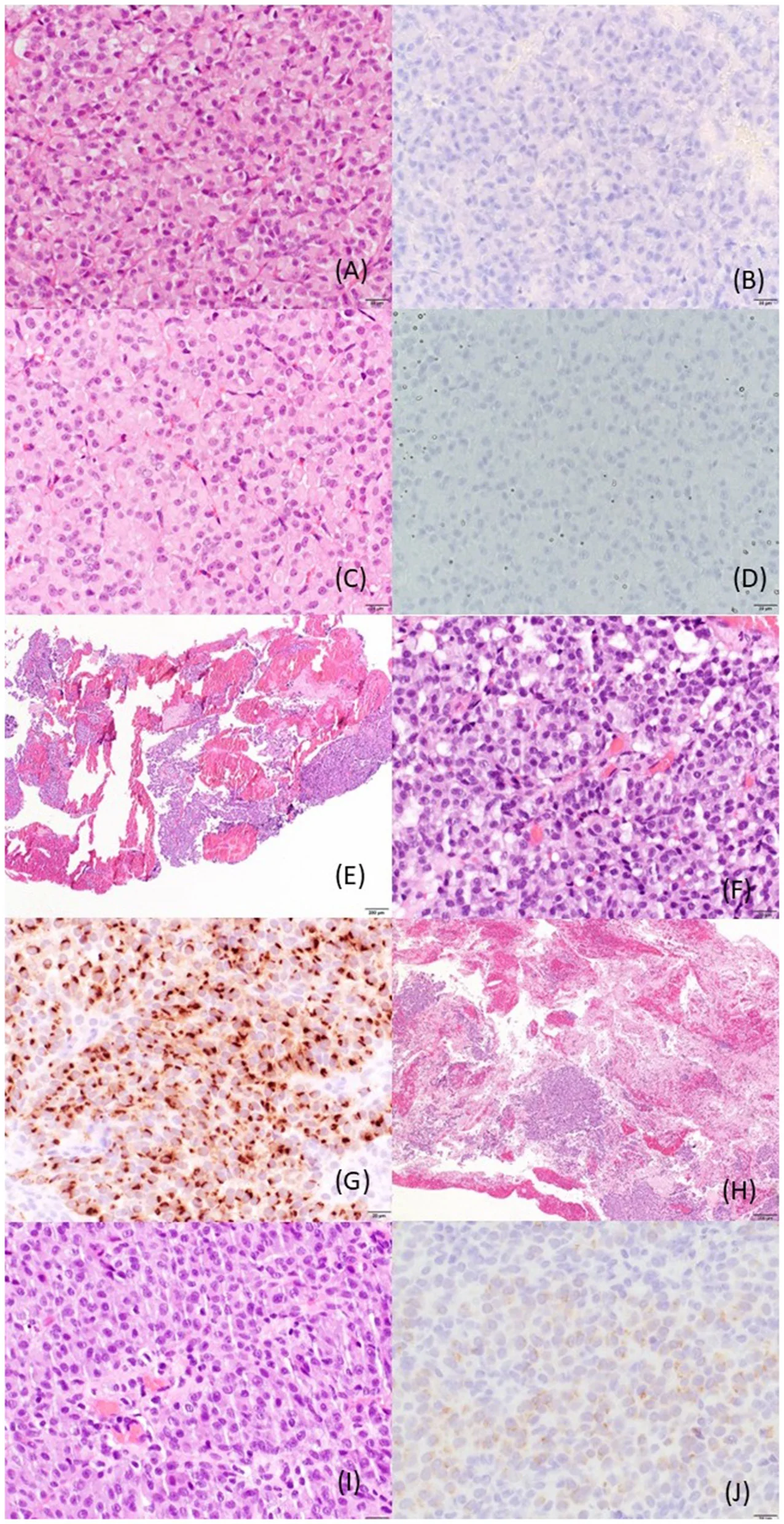
Histopathology. Histopathological studies from pituitary masses on 3 cats. **Case 1: (A)** Histology of the pituitary mass with a monotonous population of trabecula and sheets of basophilic cells. Haematoxylin and Eosin stain (H&E), 40x objective. **(B)** Negative immunohistochemistry for adrenocorticotropic hormone (ACTH) of the basophilic neoplastic cells. 40x objective. **(C)** Histology of the recurrence of the basophilic adenoma displaying a generally similar morphology as the initial sample. H&E stain, 40x objective. **(D)** The recurrence also shows negative immunohistochemistry for adrenocorticotropic hormone (ACTH) in the basophilic neoplastic cells. 40x objective. **Case 2: (E)** Histology of the pituitary mass of a cat showing variably sized fragments of basophilic cells and extensive areas of erythrocytes. The haemorrhagic aspect is consistent with apoplexy. H&E stain, 4x objective. **(F)** The neoplastic tissue consists of a moderately pleomorphic population of basophilic cells. Haematoxylin and Eosin stain (H&E), 40x objective. **(G)** Most neoplastic cells reveal moderate to marked cytoplasmic immunoreactivity for adrenocorticotropic hormone (ACTH). 40x objective. **Case 3: (H)** Histology of the pituitary mass of a cat with groups of basophilic cells and extensive areas of erythrocytes. The haemorrhagic aspect is consistent with apoplexy. H&E stain, 4x objective. **(I)** Sheets of moderately pleomorphic basophilic neoplastic cells. H&E stain, 40x objective. **(J)** A small number of neoplastic cells reveal faint to moderate cytoplasmic immunoreactivity for adrenocorticotropic hormone (ACTH). 40x objective.

Case 2 (Figures 3E–G) was also diagnosed as a basophilic adenoma. ACTH immunohistochemistry revealed strong, relatively coarse granular cytoplasmic positivity in a large number of cells. GH staining was negative within the neoplastic cell population, with only occasional scattered positively staining pre-existing adenohypophyseal cells present throughout the tissue. Alpha-MSH staining demonstrated faint background staining, likely largely nonspecific.

Case 3 (Figures 3H–J) demonstrated a basophilic adenoma with mild diffuse staining ACTH immunoreactivity and strong GH staining in a small number of scattered cells, likely representing pre-existing (normal) adenohypophyseal cells.

All 3 cases exhibited marked areas of hemorrhage, frequently paired with multifocal accumulation of pigment-laden macrophages, most consistent with siderophages, as well as cholesterol clefts, indicative of both acute and chronic hemorrhage.

## 4 Discussion

This report describes 3 cases of feline PA treated by THX, and represents, to the authors' knowledge, the first description of successful surgical management of this condition in the cat. All 3 cats were assessed at our institution under the suspicion of presence of a pituitary mass. In 2, the presenting complaint was an acute onset of neurological signs; in the third, a diagnosis of PDH had been established and neurological deficits were present. PA was retrospectively identified on histopathological examination of the pituitary glands in all 3 cases. Diagnostic imaging features of the masses were evaluated in retrospect, in search of markers for PA for future reference. A schematic overview of the clinical course of all 3 cases, from initial presentation to the most recent follow-up, is provided in Supplementary Figure 1.

In relation to the acute onset of central neurological signs, it is important to highlight that case 2 showed a positive titer IgG on both CSF and serology for Toxoplasma (T.) gondii. Notably, the IgM CSF titer was negative. T. gondii-specific antibodies can be detected in the CSF of both healthy and clinically affected cats, and may reflect either passive transfer from serum or local intrathecal production (12). The negative CSF IgM titer combined with the positive paired serum IgM titer does not support a significant intrathecal antibody production and is more consistent with a recent systemic infection or reactivation of a latent infection (12, 37). In experimental studies, cats with chronic toxoplasmosis can experience transient recurrence of IgM/IgG titer increases when the immune system is compromised (37). Given the age at presentation and the reported seroprevalence of approximately 45% in cats in Europe, prior exposure is more likely (13). Furthermore, the MRI performed at the time of the CSF sampling did not show signs of meningitis, as might be expected with acute CNS toxoplasmosis. It should be considered that blood-brain barrier disruption secondary to the apoplectic event itself may have allowed passage of serum immunoglobulins into the CSF, further complicating the interpretation of CSF antibody titres. Unfortunately, the CSF was collected prior to referral, and consequently the complete workup, including cytological evaluation, nucleated cell count, and T gondii PCR, was not available. An increase in CSF protein concentration and nucleated cell count could indicate a CNS inflammatory process; however, these findings can also occur in PA, as it can cause a chemical, sterile meningitis (14). Without paired serum or CSF titres from the same laboratory obtained at a 4-week interval demonstrating a significant rise in IgG and IgM, or a positive Toxoplasma gondii PCR on CSF, a definitive diagnosis of CNS toxoplasmosis cannot be established (37). Therefore, the T. gondii titer results from case 2 should be interpreted with caution. Nonetheless, targeted antimicrobial therapy (clindamycin) was initiated promptly following receipt of the laboratory results. The presence of an extremely large pituitary mass (P/B ratio of 1.25) and the marked improvement following surgery suggest a causal relationship between the mass and the observed clinical abnormalities. Although the clinical signs could theoretically be attributable to either pathology, the extreme size of the pituitary alone warranted decompressive surgery in the opinion of the authors. Nevertheless, a potential contribution of concurrent toxoplasmosis cannot be entirely excluded, and the impact of the infection on the clinical course of disease and outcome cannot be established with certainty.

PA is a syndrome diagnosed by combining an acute onset of neurological signs with characteristic imaging and pathological findings suggestive of hemorrhage or infarction of the pituitary gland (15–19). In human literature, it is commonly characterized by a sudden onset of severe headaches (95%−100%), followed by visual impairment, vomiting, altered levels of consciousness, and ocular palsy. These symptoms originate from a sudden increase in intrasellar pressure, caused by hemorrhage and/or infarction of the pituitary gland (18, 20, 21). Furthermore, hormonal deficiency (i.e., hypopituitarism), is also commonly reported in humans, with studies showing up to 80% of patients presenting with one or more anterior pituitary hormone deficiencies (15, 22). In canine literature, PA is described with signs of behavioral change, visual impairment, ataxia, restlessness, seizures, vestibular dysfunction (head tilt, nystagmus, vestibular ataxia) and gastrointestinal dysfunction (19, 23). In cats, episodes of restlessness, visual deficits, disorientation, and depression have been observed (4–6). The main presenting signs in our 2 clinical cases (cases 1 and 2) were restlessness with wall-hugging behavior, decreased mentation, proprioceptive deficits and hindlimb ataxia, while case 3 only presented with DM, without neurologic abnormalities. Especially the sudden onset of neurological and behavioral changes in these cases raised suspicion of PA, retrospectively. The presence of visual impairment prior to the surgical intervention in our cases is debatable. At initial presentation, case 1 did not show obvious reduction in vision, and case 2 exhibited a mildly delayed pupillary reflex and delayed visual proprioception, although there was no further evidence of blindness in the presentation of this cat. Notably, as no clinical signs of PA were demonstrated, case 3 represents a subclinical form, in which the diagnosis was made only following histopathological examination of the excised pituitary gland.

PA can be identified on advanced diagnostic imaging either on CT or MRI, where MRI is the modality of choice. The underlying pathophysiological process in PA can be ischemic infarction (i.e., with no haemorrhagic component), haemorrhagic infarction, mixed haemorrhagic infarction and clot, or a pure clot (15, 24). This helps to explain why pituitary imaging rarely shows pure infarction or hemorrhage, but rather mixed features. In dogs, characteristic CT features of acute PA include focal hyperdense areas, which can be interpreted as intratumoural hemorrhage, often combined with hypodense infarction components (25). The infarcted region usually shows no abnormalities on non-contrast imaging. Following IV contrast administration, peripheral rim enhancement may also be suggestive of PA (26). This is consistent with findings in feline cases (5, 6).

In addition to detecting the presence or absence of hemorrhage, the MRI appearance of the sellar/suprasellar mass depends on the time of onset (26). Haemorrhagic lesions on T1 and T2 weighted imaging can initially show low signal in the acute phase, while subacute lesions become hyperintense (26). This is also evident in a study on canine PA, where most dogs presented in the acute phase, rendering a hypointense T2W appearance of the pituitary lesion (23). There is also evidence that T2 demonstrates hyperintensity in the hyperacute phase, while hypointensity persists in the subacute phase (1, 22). Among the T2-weighted MRI sequences, Susceptibility-Weighted Angiography (SWAN) is particularly sensitive for the detection of haemorrhagic products. On SWAN, hemorrhage typically appears as a hypointense signal with a so-called blooming artifact, making even minimal amounts of blood products visible (11). Additionally, peripheral rim enhancement after IV gadolinium contrast administration is frequently observed on MRI in cases of PA (1, 23). Apart from modality-specific details, the prevailing opinion is, that imaging evidence of PA is characterized by a pituitary adenoma containing mixed hypo- and hyperintense areas, consistent with intratumoural hemorrhage and/or infarction.

The 3 cases presented in this manuscript demonstrated multiple findings consistent with PA on diagnostic imaging. First, it is important to recognize that although MRI is considered the gold standard for confirming PA, CT has been chosen instead of MRI in some of our cases. The initial diagnostic imaging was performed prior to referral to our clinic and depended on local availability and the preferences of the referring clinician. PA may not yet have been considered in the differential diagnoses at that stage. As the pituitary gland resides outside of the blood–brain barrier, contrast-enhanced CT can identify pituitary enlargement and can still be suggestive of haemorrhagic lesions within the gland. In case 1, as PA was confirmed on histopathology, the presence of intralesional hyperattenuating and hypoattenuating areas on CT support the interpretation that these imaging findings may have been compatible with PA. Similarly, the MRI findings of case 2 (hypointense areas within the mass) indicate compatibility with PA. The strongest evidence was provided by case 3, in which a susceptibility-sensitive MRI sequence revealed findings highly consistent with intralesional hemorrhage prior to THX. Interestingly, this patient remained free of neurological and behavioral abnormalities despite imaging evidence of pituitary hemorrhage.

In addition to the imaging findings compatible with PA, all 3 cats exhibited substantial high P/B ratios compared to those generally reported in cats with hypersomatotropism (HS), the most prevalent pituitary tumor type in this species. While pituitary enlargement is common in cats diagnosed with HS, the P/B ratio in those animals is reported to be 0.58 (9). The P/B ratios of 0.96, 1.25 and 1.12 observed in cases 1, 2, and 3, respectively, therefore reflect a substantial pituitary enlargement. Interestingly, suprasellar extension is a common finding in human and canine PA (19, 23, 27). Given these findings, the rationale for selection of surgical treatment in these cats existed; in cases in which there is a severely enlarged pituitary gland causing clinical signs, either neurological or endocrinological hyperfunction, curative intent treatment in the shape of surgical excision is generally preferred (28).

While the presence of a large pituitary mass was the primary indication for surgery in the 3 cats described in this manuscript, these cases raise interesting considerations regarding the recognition and management of feline PA. Although PA is classically regarded as a clinical syndrome, human studies have shown that up to 25% of pituitary adenomas display necrotic and/or haemorrhagic areas on diagnostic imaging (MRI), whereas clinical signs only occur in 2%−12% of patients with pituitary adenomas (29, 30). This raises the suspicion that also in feline and canine pituitary adenomas, hemorrhage and/or infarction may occur more frequently than is currently appreciated, often without causing overt clinical signs. Although well recognized in human medicine, this has not been previously described in veterinary literature (27). Case 3 appears to represent such an instance: diagnostic imaging showed findings compatible with subacute hemorrhage (Figures 1G, H), in combination with evidence of hemorrhage on histological examination, yet in absence of obvious central neurological signs. As veterinarians we must remain aware that early signs of headache and mild vision impairment are easily missed in companion animals.

The optimal therapeutic approach to PA remains controversial in human medicine. Treatment decisions are primarily based on the severity of clinical signs, in particular neuro-ophthalmic deficits and impaired consciousness (15, 31). Grading systems such as that of Jho and colleagues (27) have been proposed to support the decision-making, in particular whether immediate surgical intervention is warranted rather than elective surgery or conservative management. The rationale for weighing this urgency lies in the time-dependent nature of the outcome. Although pituitary enlargement can be temporary in PA, timely surgical decompression was advocated in the higher grades of the Pituitary Apoplexy Grading System, as these patients tended to undergo surgery earlier, while good overall outcomes were reported across all grades (27).

In current veterinary medicine, treatment choice is similarly influenced by the severity of clinical signs, although the decision has so far largely been between conservative management and euthanasia. Most reported cases, often dogs, are either managed conservatively based on clinical signs and radiological diagnosis, or euthanized and submitted for pathological confirmation when treatment is unsuccessful (19, 23). Similarly, previously reported cases of feline PA have been euthanized at the owners' request (4–6). It is likely that the lack of surgically managed cases reported in cats and dogs, is partially due to the relative lack of access to surgical treatment (28). To date, surgical treatment of PA has been described only once, in a canine patient (32). In our report, surgical therapy has been elected as the treatment of choice based on presence of either neurological complaints (case 1 and 2), or endocrinopathy (case 3), combined with the size of the pituitary found on imaging. Where previous studies have demonstrated a favorable long-term outcome following THX in cats with hormone-producing tumors (9, 33), this report further suggests that similarly favorable outcomes may also be achieved in cats with pituitary tumors complicated by concurrent PA.

Although surgery was preferred in our cases, conservative management, comprising of glucocorticoids and/or anticonvulsant medication combined with hyperosmotic agents, remains a relevant option in selected patients and has proven effective in the previously mentioned case series (19, 23). Importantly, these studies report a favorable short-term outcome, while the underlying cause of PA (i.e., pituitary mass) remains an important factor to be considered when evaluating prognosis and treatment options. The single larger cohort of dogs with PA treated with conservative management or radiation therapy, had a discouraging overall survival to discharge rate of 58% (23). Even when decompression of the mass effect might be considered a disproportionately invasive intervention, the underlying endocrine disease associated with the development of a pituitary mass, such as PDH or HS, may itself be cured by surgical intervention (9, 27, 28). Nevertheless, with the very few reports on treatment of PA in companion animals, the authors find that also conservative therapy could be considered as a possible therapeutical approach for PA.

Radiotherapy has likewise been employed in dogs with PA (19, 23). However, the effects of radiation therapy in the acute setting of PA remain controversial. In human medicine, radiotherapy has been linked to the development of apoplexy, likely due to the vascular wall damage within the tumor, caused by radiation (3, 16, 26). It is not used in the treatment of acute PA, as the effects on decreasing tumor size are slow in onset. Radiotherapy has been described as a useful approach for selected pituitary tumors in dogs, cats and humans alike (28), and has its place in the overall treatment of pituitary disease.

When navigating these treatment options, ranging from urgent surgical decompression to elective surgery, conservative management or radiotherapy, a structural framework for therapy decision-making would be beneficial. The Pituitary Apoplexy Grading System developed by Jho and colleagues could prove a valuable tool in this regard; however, it would need adaptation to the veterinary patient. In the human system, the higher grades (defined by visual impairment) typically reflect the effect of their suprasellar extension. However, the anatomy of cats and dogs differ significantly from that of humans: in these species the optic chiasm is located more rostral to the tuberculum sellae. Consequently, although visual impairment is occasionally observed, more common presentations of PA include subtle to overt changes in behavior indicative of intracranial disease (i.e., head pressing, changes in character, seeking support from walls, fear), as well as changes in ambulation and proprioception (including vestibular signs), and mentation (19, 23, 25). Furthermore, the human Pituitary Apoplexy Grading System is based upon the outcomes of a large cohort of patients (27). In veterinary medicine, the case numbers to validate an equivalent are not yet available. Altogether, with some adaptations and translational knowledge, a Feline Pituitary Apoplexy Grading System could be developed (Figure 4). The proposed system uses the severity of clinical signs as a proxy for the extent of increased intracranial pressure caused by the mass effect of the PA. It also accounts for comorbidities that affect the urgency for intervention: “modifying comorbidities” that necessitate a better stabilization prior to surgery, thus lowering the need for rapid surgical intervention, vs. “amplifying comorbidities” that may suggest that a swift intervention is preferred.

**Figure 4:**
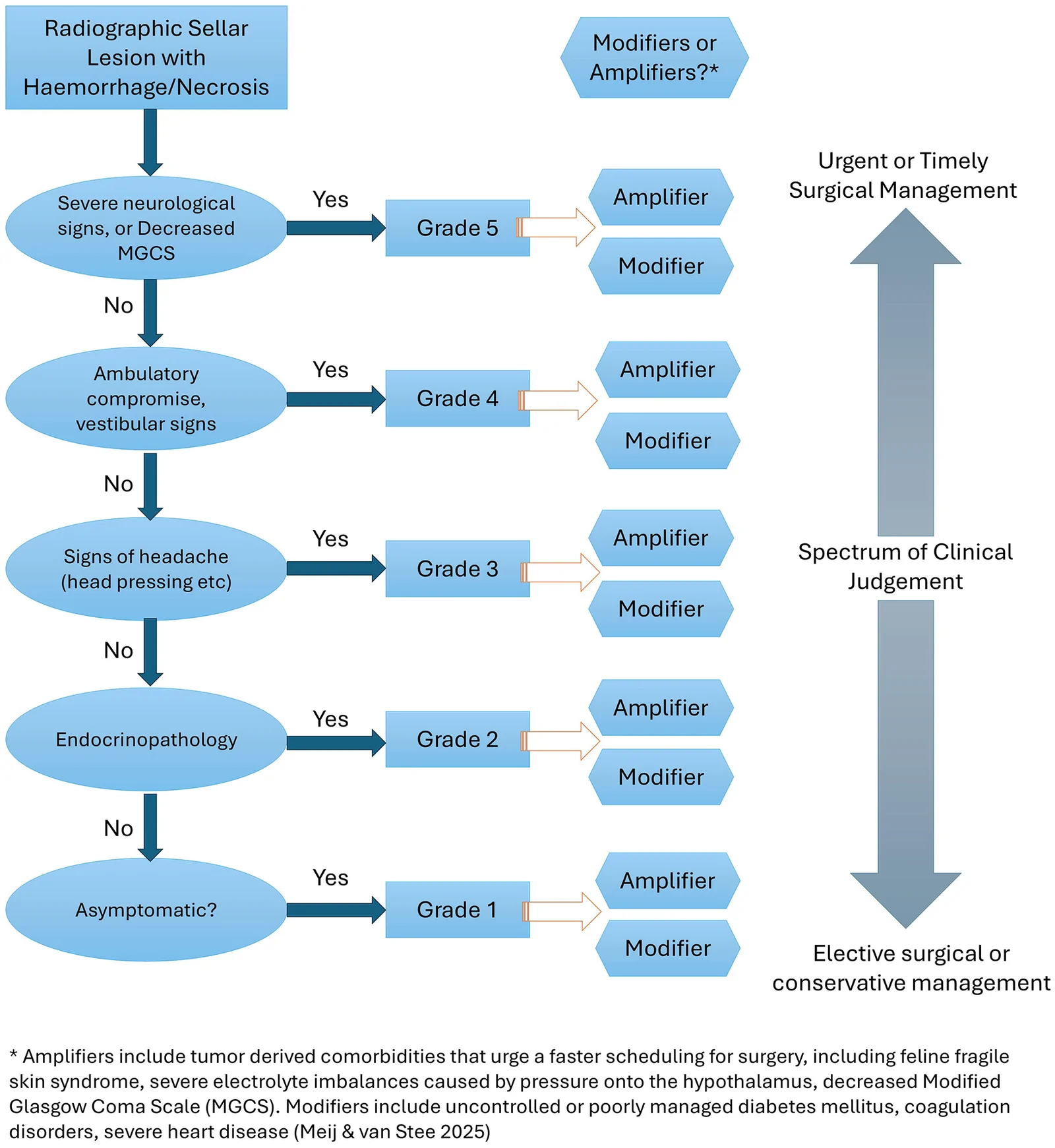
Feline pituitary apoplexy grading system. A proposed grading system for feline pituitary apoplexy was developed based on the classification described by Jho and colleagues (27). This grading system is intended to support clinical decision-making in cats with suspected pituitary apoplexy by facilitating the assessment of the urgency of intervention. Specifically, it aims to distinguish cases requiring prompt surgical management from those in which delayed elective surgery or conservative treatment may be appropriate. The grading system incorporates multiple factors, including diagnostic imaging findings, neuroendocrine and neurological clinical signs, and relevant comorbidities, all of which may influence the timing and suitability of definitive surgical treatment. *Amplifiers include tumor derived comorbidities that urge a faster scheduling for surgery, including feline fragile skin syndrome, severe electrolyte imbalances caused by pressure onto the hypothalamus, decreased modified Glasgow coma scale (MGCS) modifiers include uncontrolled or poorly managed diabetes mellitus, coagulation disorders, severe heart disease (28).

Using this proposed grading system, case 1 would be classified as a grade 3, with the behavioral abnormalities and restlessness interpreted as possible headache, which defines this grade. In this case, there were no amplifiers or modifiers that would affect the clinical judgement, placing it in an intermediate position between urgent surgical intervention and elective surgery or conservative management. Given the extremely large size of the pituitary mass, however, the author's consider elective surgical therapy an appropriate choice. Case 2 would represent grade 4 on this scale, as there was ambulatory compromise; the decreased mentation would moreover count as an amplifier, further favoring rapid surgical intervention. Case 3 would represent a grade 2 patient, as there were no neurological abnormalities. However, given the concurrent PDH, feline fragile skin syndrome and diabetes mellitus arising from the pituitary mass act as amplifiers, shifting the patient upward on the spectrum and favoring rapid surgical intervention.

Visual impairment developed postoperatively in 2 of the 3 cases of this report. In case 1 and 3, bilateral mydriasis and visual impairment were identified following THX, whereas no visual deficits had been detected at presentation. While KCS is a more frequently documented ocular complication following THX, as observed in case 1 and 2, visual impairment is not commonly reported in cats and dogs undergoing this procedure (9, 34, 35). It is conceivable that the more rostral location places the optic chiasm in closer proximity to the surgical field during THX compared to humans, rendering it more vulnerable to iatrogenic injury (36). The absence of both the menace response and pupillary reflexes in case 1, without detectable intraocular abnormalities, could be consistent with injury to the optic nerves or optic chiasm. Case 3 did show lack of pupillary reflexes, combined with presence of both menace response and dazzle reflexes in both eyes and no abnormalities found on fundoscopy. Mydriasis became less pronounced over time and vision partially recovered over time. The most likely explanation would be the same as in case 1; extraocular optic neuritis, likely related to the THX. The initial classification of cortical blindness in case 3 was likely due to concurrent sudden behavioral changes of the cat, and the presence of both menace response and dazzle reflex. Mydriasis was attributed to the opioid administration, however, mydriasis persisted after ceasing opioid therapy. The positive menace reflex could also be caused by airflow and subsequent corneal reflexes and is likely overinterpreted at the time. In both cases, fundoscopy did not show evidence of optic neuritis, but lack of intraocular changes to the optic disc does not exclude extraocular optic neuritis. The optic nerves and optic chiasm reside relatively rostral to the pituitary fossa, however, in large tumors, surgical excision may still evoke local damage as large masses can expand rostrally and thus, extraction necessitates rostral tumor manipulation. The visualization on the rostral border of the pituitary fossa, beyond the tuberculum sellae, is poor, and inadvertent manipulation of structures rostral to the burr hole is possible. Further improvement of intraoperative visualization techniques including endoscopy may prevent iatrogenic trauma and improve outcomes in these cases.

Repeated follow-up imaging at 6- to 12-month intervals was recommended in all 3 cases to identify presence of tumor recurrence. This interval was based on data from previous cohorts of cats with pituitary tumors that underwent THX. A study by Meij et al. (34) reported tumor recurrence in a cat with PDH after 19 months, which was diagnosed based on clinical and biochemical signs of hyperadrenocorticism. Additionally, a median recurrence time of 248 days (range 84–1,232 days) was reported in 5 cats with hypersomatotropism (33). In case 1, the CT imaging performed 3 months postoperatively revealed changes within the hypophyseal fossa that were potentially consistent with a small amount of residual hypophyseal tissue, although these findings were not conclusive. Given the definitive evidence of tumor recurrence on imaging approximately 2 years after surgery, it remains possible that microscopic residual tissue persisted following the initial surgical intervention. The owner of cat 2 declined further imaging. In case 3, intraoperative inspection of the fossa showed no abnormal tissue present, but the direct postoperative CT scan did show a small area of contrast enhancement indicative of possible remnant pituitary tissue. CT scan at 1 year post surgery showed recurrence of a mass in the pituitary fossa, although this time, no recurrence of PDH was evident in the UCCRs and the cat remained without clinical signs. This illustrates the difficult balance between achieving complete tumor resection, while preserving normal hypothalamus tissue, as visualization of the surgical field in this region is inherently limited. Endoscopic inspection, which is now routinely performed following THX at this facility, appears to enhance visualization of the hypophyseal fossa, and may facilitate the identification of residual pituitary tissue, although its use remains an area for future research.

## 5 Conclusion and future perspectives

PA should be considered a differential diagnosis in cats presenting with an acute onset of neurological signs, particularly when there is evidence of an enlarged sellar mass on pituitary imaging. The presented cases demonstrate that THX can offer a positive outcome in cats with PA. For future studies, a more systematic assessment of PA on advanced imaging is warranted to better determine the frequency of hemorrhage and infarction in association with pituitary tumors. Additionally, closer alignment between the surgical, imaging and pathology disciplines will lead to more consistent diagnoses of this syndrome.

## Data Availability

The raw data supporting the conclusions of this article will be made available by the authors, without undue reservation.
